# Examination and Relationship of Posterior Superior Alveolar Artery and Canalis Sinuosus Using Cone Beam CT

**DOI:** 10.3390/biomimetics10060352

**Published:** 2025-06-01

**Authors:** İskender Yılmaz, Sevda Lafci Fahrioglu, Mujgan Firincioglulari, Kaan Orhan, Sezgin İlgi

**Affiliations:** 1 Department of Anatomy, Faculty of Medicine, Near East University, Nicosia 99138, Cyprus; sezgin.ilgi@neu.edu.tr; 2 Department of Anatomy, Faculty of Medicine, University of Kyrenia, Kyrenia 99000, Cyprus; 3 Department of Anatomy, Faculty of Medicine, Cyprus Health and Social Sciences University, Mersin 10 Turkey, Morphou 99750, Cyprus; sevdalafci@gmail.com; 4 Department of Dentomaxillofacial Radiology, Faculty of Dentistry, Cyprus International University, Nicosia 99010, Cyprus; mujganfirincioglulari@gmail.com; 5 Department of Dentomaxillofacial Radiology, Faculty of Dentistry, Ankara University, Ankara 06560, Turkey; knorhan@dentistry.ankara.edu.tr

**Keywords:** posterior superior alveolar artery, canalis sinuosus, cone beam computed tomography, maxilla, anatomy

## Abstract

In this study, we investigated the anatomical location, dimensions, and relationships of the posterior superior alveolar artery (PSAA) and canalis sinuosus (CS) within the maxilla, aiming to enhance the safety and efficacy of surgical procedures. A retrospective analysis was performed on 323 individual cone beam computed tomography scans. The diameter of the PSAA and CS, the distance of the PSAA from the sinus floor, the distance of the PSAA and CS from the alveolar crest, the distance of the PSAA and CS from the nasal septum, and the distance from CS to the nasal cavity floor were measured. The distance between PSAA and the sinus floor showed no significant difference between the right and left sides nor between genders (*p* < 0.05). The distance between the alveolar crest of PSAA and the distance between PSAA and to nasal septum was significantly higher on the left than on the right side (*p* < 0.05). According to gender, female subjects exhibited a lower distance between PSAA and the nasal septum than males (*p* < 0.05). Variations in PSAA and CS anatomy highlight the need for individualized preoperative CBCT assessment to reduce complications like bleeding during maxillary surgeries, enhancing surgical planning and safety in dental and maxillofacial procedures.

## 1. Introduction

Over the past decade, there has been a notable increase in surgical interventions for superior repositioning of the maxilla, such as total maxillary alveolar osteotomy, Caldwell–Luc surgery, oral implant placement, Le Fort I osteotomy, and bone grifting procedures; parallel to increasing in maxillary surgeries, the frequency of intraoperative and postoperative complications has also risen. Although significant nerves and vessels, such as the nasopalatine artery and nerve, follow a relatively stable course in the anterior maxilla, the course of thinner vessels and nerves, such as the posterior superior alveolar artery (PSAA), may vary. It is necessary to examine the neurovascular structures in the maxilla thoroughly [1,2].

Maxilla is one of the visceral bones, and it is called the upper jaw bone [3]. The anterior, middle, and posterior superior alveolar arteries are the primary arteries responsible for the vascular supply of the maxilla [4]. The PSAA, a branch of the maxillary artery, typically originates from its third segment. It descends downwards along the infratemporal face of the maxilla. It divides into two branches before passing through the posterior superior alveolar foramen. Both branches connect with the infraorbital artery (IOA), forming an intraosseous and extraosseous loop. It plays a key role in maxillary vascularization. This anastomotic network runs along the maxillary sinus’s lateral wall. It supplies blood to the sinus membrane (Schneiderian membrane) and the periosteal tissues beneath it, particularly the anterior and lateral wall of the maxillary sinus [5,6]. Traumas in this region can damage the anastomotic network, leading to severe bleeding in the mucosal layer of the maxillary sinus [3].

The IOA, originating from the same trunk as the PSAA, runs along the sinus roof, giving off branches to anastomose with the branches of the PSAA. Before exiting the infraorbital foramen, it gives rise to the anterior superior alveolar artery. This branch travels within the canalis sinuosus (CS) and opens into the roof of the mouth, posterior to the canine teeth. For esthetic reasons or rehabilitation purposes, the anterior aspect of the maxilla is frequently subjected to various surgical procedures due to trauma or tooth loss [7,8]. The variations and anatomical positions of these neurovascular structures, essential in dental surgery, continue to be discussed today [9,10].

During procedures involving the paranasal sinus region, knowing that the anastomosis of the PSAA and the IOA is at risk is crucial. The likelihood of intraoperative bleeding is linked to the artery’s diameter—the larger the artery, the higher the risk of severe hemorrhage. The PSAA is of great clinical significance because it is included in many surgical procedures [11,12].

Cone beam computed tomography (CBCT), one of the modern imaging techniques, allows for the three-dimensional, reproducible evaluation of the location, size, and relationship of structures with others while exposing the patient to less radiation [13,14].

In our study, we aimed to provide insights for clinicians performing surgical interventions by examining the PSAA and CS morphological characteristics and their relationship with surrounding structures in the Cypriot population. We also aim to understand maxillary vascular anatomy while exploring the spatial relationships of these neurovascular structures with surrounding maxillary landmarks relevant to surgical planning. Additionally, we provide population-specific anatomical data that may aid in pre-surgical risk assessment and personalized treatment planning. Through these evaluations, we intend to assist clinicians in minimizing the risk of intraoperative complications and enhancing surgical outcomes during maxillary procedures.

## 2. Materials and Methods

### 2.1. Study Design

The research received approval from the Research and Ethics Committee (EKK23-24/006/08) based on the principles of the 1964 Helsinki Declaration, its later revisions, or comparable ethical standards. It also followed the ethical guidelines established by institutional and national research committees for procedures involving human participants.

A total of 323 randomly selected CBCT scans of individuals who visited the dental imaging center for various reasons were retrospectively analyzed. The CBCT images included in the study were obtained from individuals aged over 18. Scans with high image quality, free of artifacts, and displaying the maxillofacial planes were included in the study. The exclusion criteria were as follows: images with poor diagnostic quality due to motion or metal artifacts; the presence of impacted teeth, retained roots, or dental implants; cases involving syndromes affecting the dentomaxillofacial region (e.g., cleft lip and palate); foreign bodies in the area of interest that could interfere with the assessment of CS; the presence of metabolic, infectious, or tumor lesions in the maxillary region; patients undergoing orthodontic treatment or with a history of previous surgical procedures or trauma to the anterior maxilla.

### 2.2. CBCT Imaging Acquisition

CBCT imaging was performed using the Newtom GO 3D/2D system (Quantitative Radiology s.r.l., Verona, Italy). The scanning parameters were set to 90 kVp, 4 mA, a 24 s exposure time, a voxel size of 0.3 mm, and a 10 × 10 cm field of view.

### 2.3. Anatomical Background and Radiological Identification

The posterior superior alveolar artery, a branch of the maxillary artery, is essential for supplying blood to the maxillary sinus and its surrounding structures. CBCT imaging effectively visualizes the PSAA, which typically appears as a hypodense canal situated within or near the lateral wall of the maxillary sinus [15].

The canalis sinuosus is a neurovascular canal that transports the anterior superior alveolar nerve and vessels, stretching from the infraorbital canal to the anterior maxilla. CBCT imaging provides clear visualization of the CS, which is seen as a narrow canal in both sagittal and axial views, frequently located near the apices of the anterior teeth [16].

### 2.4. Radiological Assessment

**Figure 1:**
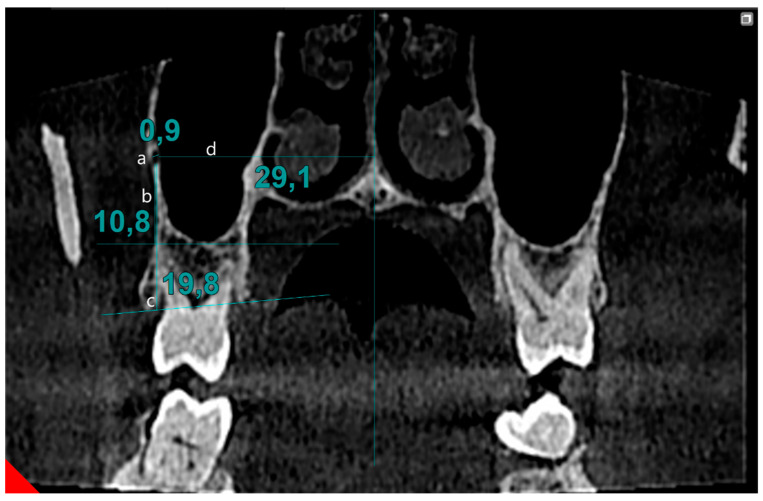
Evaluation of PSAA parameters: (**a**) diameter of PSAA, (**b**) distance of PSAA from sinus floor, (**c**) distance of PSAA from alveolar crest, (**d**) distance of PSAA from midline.

Cone beam computed tomography data were assessed by two observers (M.F. and İ.Y.) on two separate occasions, with a two-week interval between evaluations, to assess inter- and intra-observer variability. Scans were performed in the coronal section, moving from posterior to anterior, to identify the PSAA canal. During the scanning process, if a canal in the lateral wall of the sinus was detected, its image in the transverse section was simultaneously reviewed. Once confirmed, measurements of the canal continued in the coronal section. The localization of the canal was determined based on its position in the bone tissue: extraosseous (outside the bone), intraosseous (within the bone), and intranasal (inside the maxillary sinus). The image was magnified fourfold to determine the canal diameter. The line drawn between the inner surfaces of the cortical bones was defined as the canal’s diameter. The distance to the sinus floor and alveolar crest was measured from the canal. Also, the distance between the nasal septum and the central point of the canal was recorded (Figure 1).

**Figure 2:**
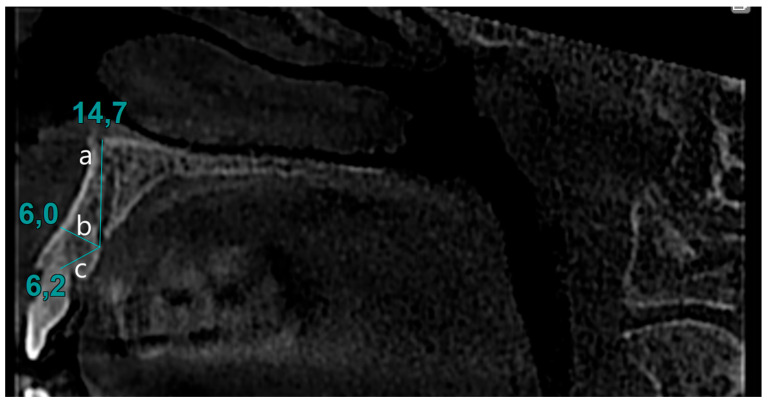
Evaluation of CS parameters: (**a**) distance to nasal cavity floor from CS, (**b**) distance to buccal bone from CS, (**c**) distance to alveolar crest from CS.

The presence of the CS in the sagittal section was identified. The identified images were verified in the transverse section. Diameters of CS were calculated by measuring the opening at the palatal cortical plate. The distance between the canal opening and the tangent line drawn to the nasal cavity floor was measured in the sagittal section. Similarly, the distance between the canal and the alveolar crest, as well as the distance between the buccal cortical bone and the CS, was determined using the same method (Figure 2).

**Figure 3:**
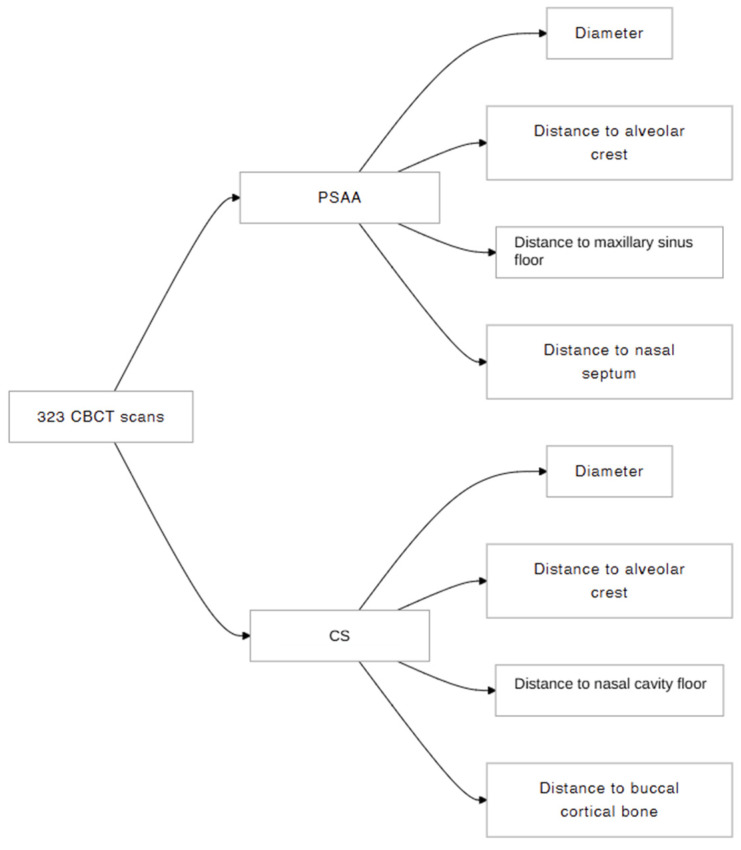
Research diagram flow of this study.

Figure 3 illustrates the research diagram flow of the present study.

### 2.5. Statistical Analysis

Within the scope of the study, the data obtained from PSAA and ASA observations of 323 patients—147 females and 176 males aged between 18 and 93 years—were defined by entering data into the SPSS 24 data analysis program. After the data entry was defined, frequency and percentage distributions of the distributions related to the individual characteristics and measurements of the patients, as well as valid percentage distributions excluding missing data, were determined.

In addition to calculating the mean ($\bar{x}$) and standard deviation (σ) values of the PSAA and CS measurements observed in the right and left jaws, descriptive analyses were performed to determine the Skewness (β_1_) and Kurtosis (β_2_) values to determine the normal distribution. In determining the normal distribution, the normal distribution ranges of the Skewness and Kurtosis calculations were utilized. Thus, for variables with Skewness (β_1_) and Kurtosis (β_2_) values within a range of ±1.5, independent groups *t*-test, paired samples *t*-test, and one-way ANOVA analysis drawn from parametric statistics were used, while for β_1_ and β_2_ values above ±1.5, the Mann–Whitney U test drawn from non-parametric tests was used. For gender comparisons, the significance level was determined by independent sample *t*-test and Mann–Whitney U analysis. One-way ANOVA analysis was used for age comparisons, and Bonferroni test was used to make post hoc pairwise comparisons between significantly different results since variance homogeneity was ensured. This test was preferred as it provides more reliable results in intergroup comparisons due to its tendency to reduce false positive results and the Type 1 error rate. Within the scope of the study, cross-tabulation analysis and Pearson χ2 (chi-square) analysis were used to determine the cross-distributions. Thus, the extent to which the comparisons created a significant difference was determined.

Both intra- and inter-examiner reliability were evaluated. The Wilcoxon signed-rank test assessed intra-observer consistency, while inter-observer reliability was verified through an intraclass correlation coefficient (ICC > 0.75) and a low coefficient of variation, demonstrating high reproducibility.

## 3. Results

In this study, data were obtained from 323 individuals. Out of 323 individuals, 300 (92.9%) patients had PSAA on the right side, and 291 (90%) patients had PSAA on the left side. Moreover, 271 (83.9%) patients showed bilateral PSAA, while 49 (15.2%) patients showed unilateral PSAA.

Also, out of 323 patients, 138 (42.7%) had CS on the right side and 127 (39.3%) had it on the left side. In terms of unilateral and bilateral CS, 101 (31.3%) patients had unilateral CS and 82 (25.4%) patients had bilateral CS. Table 1 shows the gender and age distributions and anatomical characteristics of PSAA and CS.

The mean diameter of the right PSAA was $\bar{x}$ = 0.82 ± 0.22, while the left PSAA measured $\bar{x}$ = 0.84 ± 0.25. The mean distance between PSAA and the sinus floor on the right side was $\bar{x}$ = 9.66 ± 3.59, while the mean of the left side was $\bar{x}$ = 9.99 ± 3.41. (*p* > 0.05) The mean distance between the alveolar crest and PSAA was found to be $\bar{x}$ =18.17 ± 3.28 on the right side and $\bar{x}$ =18.67 ± 3.08 on the left side. The distance of PSAA on the left side was statistically significantly higher than on the right side (*p* < 0.05). The distance of PSAA to the nasal septum was found to be $\bar{x}$ = 30.35 ± 2.13 on the right side and $\bar{x}$ = 30.64 ± 2.16 on the left side. The distance of PSAA to the nasal septum on the left side was statistically higher than on the right side (*p* < 0.05). Morphometric parameters were analyzed in the presence of bilateral PSAA (Table 2).

For CS, the mean right diameter was 0.79 ± 0.26, and the left diameter was 0.83 ± 0.28. (*p* > 0.05) The mean distance of CS to the nasal cavity floor on the right side was 13.47 ± 2.73, and the mean on the left was 13.20 ± 2.83 (*p* > 0.05). In CS, the mean distance to the alveolar crest was $\bar{x}$ = 5.95 ± 2.05 on the right side and $\bar{x}$ = 6.25 ± 2.55 on the left side. (*p* > 0.05). The mean distance between CS and buccal bone margin was 7.04 ± 1.82 on the left side and 7.20 ± 1.87 on the right side (Table 3).

When the distance of PSAA to the nasal septum was analyzed according to gender, it was found that female individuals had a mean of 30.06 ± 2.03 on the left side, which was 31.06 ± 2.12 for male individuals on the same side. There was a statistically significant difference (*p* < 0.005). On the right side, female individuals had a mean of 29.99 ± 2.19, while male individuals had a mean of 30.61 ± 2.08. Female individuals showed statistically significantly lower values than males (*p* < 0.005). (Table 4). CS and gender comparisons are shown in Table 5, and there was no significant difference found.

Correlation analysis was performed between the PSAA and CS parameters (alveolar crest and diameter), and no significant difference was found (*p* > 0.05).

Significant differences in this study highlight the importance of considering these parameters, particularly the distance between PSAA and critical anatomical structures such as the maxillary sinus floor, alveolar crest, and nasal septum, in the surgical planning of sinus lifts or dental implants.

## 4. Discussion

It is known that PSAA and CS contain arterial structures of the same origin [6]. Although they are evaluated separately in studies, both structures have crucial importance in oral procedures. Knowing the anatomical position of these structures in oral procedures, especially in terms of bleeding risk, is very important to minimize complications in surgical procedures [11,12]. In our study, we aimed to evaluate the anatomical position of the structures (PSAA and CS) and their relationship with each other in the Cypriot population.

The PSAA was identified in 99.1% of the patients, with the majority being intraosseous (74.5%). Godil et al. [17] identified PSAA in 99.4% of the patients; Shahidi et al. [18] found an identification rate of 93%, resembling our results. On the other hand, our success rate for identifying the artery exceeded the rates reported by Hayek et al. (50%) [19], Keceli et al. (49.8%) [20], and Lozano et al. (48.6%) [21]. This difference can be attributed to differences in the ethnicity of the subjects and sample sizes. Danesh et al. [11] and Ilgüy et al. [22] also stated that intraosseous-type PSAA had the highest percentage.

The location of CS is mostly seen at the central incisors (50.2%) and followed by the lateral incisor (37%) region. Our results resemble the findings of Samunahmetoglu et al. [23] and Arx et al. [24]. They found the highest frequency of occurrence at the central incisors, followed by the lateral incisors.

The mean right-sided diameter obtained from 300 PSAA images was 0.82 ± 0.22 mm, and the mean left-sided diameter was 0.84 ± 0.25 mm for 291 patients. No significant difference was found between the right and left sides (*p* < 0.05). Resembling our results, Encina et al. [25] reported that in their study of 212 patients, the PSAA diameter was 1.1 ± 0.4 mm on both sides; Rathod et al. [3] reported that in 150 patients, the diameter on the left side was 1.30 ± 0.42, which was 1.19 ± 0.40 higher than the diameter on the right side, and they found no significant difference. We believe that the difference in PSAA diameter from that of other studies [3,25] is associated with each study’s sample size, which was smaller than ours.

In this study, the diameter of CS is reported as 0.79 ± 0.26 mm on the right side and 0.83 ± 0.28 mm on the left side. Sun et al. [26] reported the mean diameter of the CS as 0.89 ± 0.26 mm, while Devathambi et al. [10] stated that the average diameter was 0.81 ± 0.21 mm on the right side and 0.83 ± 0.26 mm on the left side, similar to our results. These findings have been supported by Aoki et al. [27], who reported that 96% of the CS measurements were less than 1 mm.

The distance from the PSAA to the sinus floor was found to be $\bar{x}$ = 9.66 ± 3.59 mm on the right side and $\bar{x}$ = 9.99 ± 3.41 mm on the left side, and no significant difference was found (*p* > 0.05). Tassoker et al. [28] reported that these values were 9.25 ± 3.74 mm and 8.83 ± 3.79 mm, respectively, with no significant difference. Moreover, Haghanifar et al. [29] did not find a significant difference between the distance of PSAA to the sinus floor on the right and left sides. Karslioglu et al. [30] reported this value as 10.37 ± 3.98 mm in their study. However, studies in which this value was lower have been reported in the literature [31,32].

Our results showed that there was a significant gendered difference between the distances of the PSAA and the maxillary sinus floor (*p* < 0.05). Male patients exhibited a greater distance than female patients. Çam et al. [33], Güncü et al. [34], and Ilgüy et al. [22] found that the distance between PSAA and maxillary sinus floor was found to be statistically significantly greater in males, resembling our result.

The distance between PSAA and the alveolar crest was found to be $\bar{x}$ = 18.17 ± 3.28 mm on the right side and $\bar{x}$ = 18.67 ± 3.08 mm on the left side in our study, with a significant difference (*p* < 0.05). Rathod et al. [3] reported that this distance varies depending on the localization of the PSAA. Güler et al. [35] and Kale et al. [36] reported no significant difference between the distance of PSAA to the alveolar crest.

Although our study’s results are compatible with the average values in the literature, we believe that the different results reported are due to the location of the PSAA, supporting the findings reported by Rathod et al. [3]. These differences in distance measurements across studies could be attributed to anatomical variations in the positioning of the arteries.

Tehranchi et al. [37] found a significant difference between males and females in terms of PSAA-to-nasal-septum distance; more specifically, it was higher in males. Likewise, our results showed that the distance of female individuals to the nasal septum was statistically lower than that of male individuals. On the other hand, the distance from PSAA to the nasal septum was found to be $\bar{x}$ = 30.64 ± 2.16 mm on the left side and $\bar{x}$ = 30.35 ± 2.13 mm on the right side. Results showed that the left side was statistically significantly higher than that on the right side (*p* < 0.05). To the best of our knowledge, there is no previous study that compares distances between PSAA and the nasal septum on both the left and right sides.

The distance from the CS to the nasal cavity floor was 13.47 ± 2.73 mm on the right side and 13.20 ± 2.83 mm on the left side. Devathambi et al. [10] reported it as 15.86 ± 3.14 mm for the right side and 16.26 ± 3.33 mm for the left side. To our knowledge, no other study has been found in the literature that compares the right and left sides of the CS. As previously mentioned, we believe that the differences in our values are attributable to population variations. The PSAA values within the same population are relatively consistent, and there are insufficient studies available for a comprehensive comparison of CS.

The distance between CS and the alveolar crest was found to be $\bar{x}$ = 5.95 ± 2.05 mm on the right side and $\bar{x}$ = 6.25 ± 2.55 mm on the left side. In the literature, Sun et al. reported the mean CS to alveolar crest distance as 5.78 ± 2.25 mm [26]; while Rao et al. [38] reported the mean CS to alveolar crest distance as 12.207 ± 3.809 mm in male subjects and 10.829 ± 3.080 mm in female subjects.

The limitation of this study may be the small sample size; therefore, further research in this area with larger samples is recommended.

When the correlations of PSAA and CS for the same parameters on the left and right sides were analyzed, no statistically significant correlation was found. In addition, these correlations have not been previously analyzed in the literature. No significant correlation was found between PSAA and CS in the Cypriot population.

Given these results, it is recommended that clinicians consider population-based averages for the position and size of PSAA and CS to improve surgical accuracy. The assessed parameters provide detailed information on the morphology and location of these structures, including their distances from surrounding anatomical landmarks. This knowledge can help surgeons better understand these variations during preoperative planning, enabling more precise surgical approaches and reducing the risk of complications in both the preoperative and postoperative stages.

## 5. Conclusions

The study found no significant difference in the diameter of PSAA between the right (0.82 ± 0.22 mm) and left (0.84 ± 0.25 mm) sides (*p* > 0.05). However, the left side had a significantly greater distance to the maxillary sinus floor, alveolar crest, and nasal septum (*p* < 0.05). For CS, no side-to-side differences in diameter (right: 0.79 ± 0.26 mm, left: 0.83 ± 0.28 mm) were found (*p* > 0.05). Females showed smaller PSAA diameters and shorter distances to the nasal septum than males, while no gender differences were found for CS. In conclusion, CBCT is valuable for preoperative assessment of PSAA and CS, aiding in surgical planning for dental procedures. Future studies with larger sample sizes could offer more insights into these anatomical variations.

## Figures and Tables

**Table 1:** Anatomical characteristics of PSAA and CS, gender, and age distributions.

		*f*	%	Valid %
Gender	Female	147	45.5	45.5
	Male	176	54.5	54.5
Age	<30.0	51	15.8	15.8
	31.0–50.0	93	28.8	28.8
	51.0–70.0	142	44.0	44.0
	71.0+	37	11.5	11.5
Right PSAA Type	Intraosseous	234	72.4	78.0
	Extraosseous	41	12.7	13.7
	Intrasinusal	25	7.7	8.3
	Total Observed	300	92.9	100.0
	Missing	23	7.1	
Left PSAA Type	Intraosseous	212	65.6	72.9
	Extraosseous	70	21.7	24.1
	Intrasinusal	9	2.8	3.1
	Total Observed	291	90.1	100.0
	Missing	32	9.9	
Septum Deviation	No	202	62.5	62.5
	Yes	121	37.5	37.5
Right CS Location	Central incisor	62	19.2	44.9
	Lateral incisor	58	18.0	42.0
	1st premolar	11	3.4	8.0
	Canine	7	2.2	5.1
	Total Observed	138	42.7	100.0
	Missing	185	57.3	
Left CS Location	Central incisor	71	22.0	55.5
	Lateral incisor	41	12.7	32.0
	1st premolar	11	3.4	8.6
	Canine	5	1.5	3.9
	Total Observed	128	39.6	100.0
	Missing	195	60.4	
Total Patients		323	100.0	

**Table 2:** Comparison of PSAA morphometric parameters according to sides. * shows statistical significance. (*p* < 0.05).

PSAA			
	Left (n = 271)	Right (n = 271)	*p*
	Mean ± SS Median (Min–Maks)	Mean ± SS Median (Min–Maks)	
Diameter	0.84 ± 0.25 0.80 (0.3–2.1)	0.82 ± 0.22 0.80 (0.3–1.5)	0.690
Distance to maxillary sinus floor	9.99 ± 3.41 9.90 (1.8–18.9)	9.66 ± 3.59 9.60 (1.2–19.2)	0.087
Distance to alveolar crest	18.67 ± 3.08 18.90 (9.3–27.3)	18.17 ± 3.28 18.30 (8.4–27.6)	0.013 *
Distance to nasal septum	30.64 ± 2.16 30.60 (25.5–37.9)	30.35 ± 2.13 30.30 (25.5–36.9)	0.002

**Table 3:** Comparison of CS morphometric parameters according to sides. *p* < 0.05 shows statistical significance.

CS			
	Left (n = 82)	Right (n = 82)	*p*
	Mean ± SS Median (Min–Maks)	Mean ± SS Median (Min–Maks)	
Diameter	0.83 ± 0.28 0.70 (0.4–1.6)	0.79 ± 0.26 0.70 (0.4–1.6)	0.127
Distance to nasal cavity floor	13.20 ± 2.83 13.40 (6.3–20.3)	13.47 ± 2.73 13.65 (6.3–18.8)	0.464
Distance to buccal bone	7.04 ± 1.82 6.90 (3.0–13.5)	7.20 ± 1.87 7.10 (1.5–13.8)	0.538
Distance to alveolar crest	6.25 ± 2.55 5.55 (2.6–13.4)	5.95 ± 2.05 5.70 (1.6–12.4)	0.743

**Table 4:** Comparison of PSAA parameters in terms of gender. *p* < 0.05 shows statistical significance. * shows statistical significance (p<0.05).

	Female (n = 140)	Male (n = 160)	*p*-Value
	Mean ± SD Median (Min–Max)	Mean ± SD Median (Min–Max)	
**Right PSAA diameter**	0.81 ± 0.22 0.75 (0.4–1.5)	0.82 ± 0.21 0.80 (0.3–1.5)	0.481
Right PSAA to maxillary sinus floor distance	9.32 ± 3.67 9.00 (1.2–18.6)	9.91 ± 3.51 9.90 (2.7–19.2)	0.114
Right PSAA to alveolar crest distance	18.08 ± 3.40 18.00 (10.8–27.6)	18.29 ± 3.26 18.60 (8.4–25.8)	0.447
Right PSAA to nasal septum distance	29.99 ± 2.19 30.00 (25.5–35.7)	30.61 ± 2.08 30.60 (25.5–36.9)	0.009 *
	Female (n **= 133**)	Male (n **= 158**)	
	Mean ± SD Median (Min–Max)	Mean ± SD Median (Min–Max)	*p*-value
Left PSAA diameter	0.82 ± 0.27 0.80 (0.3–2.1)	0.84 ± 0.23 0.85 (0.4–1.7)	0.371
Left PSAA to maxillary sinus floor distance	9.17 ± 3.55 9.00 (1.8–18.3)	10.58 ± 3.15 10.50 (3.9–18.9)	<0.001 *
Left PSAA to alveolar crest distance	18.31 ± 3.51 18.60 (9.3–27.3)	18.84 ± 2.85 19.20 (8.7–26.4)	0.179
Left PSAA to nasal septum distance	30.06 ± 2.03 30.00 (25.5–34.8)	31.06 ± 2.12 30.90 (26.4–37.9)	<0.001 *

**Table 5:** Comparison of CS parameters in terms of gender. *p* < 0.05 shows statistical significance.

	Female (n = 57)	Male (n = 81)	
	Mean ± SD Median (Min–Max)	Mean ± SD Median (Min–Max)	*p*-value
**Right CS diameter**	0.75 ± 0.25 0.70 (0.4–1.3)	0.81 ± 0.28 0.70 (0.4–1.6)	0.256
Right CS to nasal cavity floor distance	13.36 ± 2.58 13.60 (6.3–18.8)	13.56 ± 2.94 13.80 (6.3–18.8)	0.548
Right CS to buccal bone distance	6.90 ± 1.66 6.80 (1.5–12.2)	7.13 ± 1.76 7.00 (2.8–13.8)	0.475
Right CS to alveolar crest distance	6.17 ± 2.51 5.70 (1.6–15.1)	6.61 ± 2.25 6.20 (2.8–14.3)	0.136
	Female (n **= 65**)	Male (n **= 62**)	
	Mean ± SD Median (Min–Max)	Mean ± SD Median (Min–Max)	
Left CS diameter	0.82 ± 0.28 0.70 (0.4–1.6)	0.80 ± 0.26 0.70 (0.4–1.5)	0.842
Left CS to nasal cavity floor distance	12.96 ± 3.06 13.50 (4.8–19.0)	13.48 ± 2.54 13.55 (7.5–20.3)	0.526
Left CS to buccal bone distance	7.11 ± 1.75 6.80 (3.0–13.5)	6.74 ± 1.54 6.80 (2.8–9.9)	0.376
Left CS to alveolar crest distance	6.60 ± 2.86 5.80 (2.6–13.4)	6.19 ± 2.43 5.55 (2.7–14.1)	0.556

## Data Availability

The datasets used and/or analyzed during the current study are available from the corresponding author upon reasonable request.
